# The Moderating Role of Intellectual Humility in the Adoption of ICT: A Study Across Life-Span

**DOI:** 10.3389/fpsyg.2018.02433

**Published:** 2018-12-04

**Authors:** Gloria Bernabé-Valero, Isabel Iborra-Marmolejo, Maria J. Beneyto-Arrojo, Nuria Senent-Capuz

**Affiliations:** Department of Occupational Sciences, Speech Therapy, Evolutionary Psychology and Education, Catholic University of Valencia San Vicente Mártir, Valencia, Spain

**Keywords:** intellectual humility, ICT, age, cognitive variables, thinking dispositions

## Abstract

Literature has shown age-related differences in the adoption process. In this way, it is very important to encourage the adoption of ICT by the elderly, in order to maintain their independence in daily life. However, some specific cognitive variables were not considered in theoretical models until a decade ago. One of the emerging fields in this area is the science of learnable intelligence, which investigates the role of thinking dispositions. The variable which we have focused on is intellectual humility. For this propose, a sample of 306 participants from 18 to 87 years was selected. Age was selected as a predictor variable. Intellectual humility was tested as a moderator between aging and ICT adoption, more precisely computer and mobile devices. The model fitted the theoretical proposal. However, the subscale known as Independence of the intellect and ego was the only one to fulfill all the requirements for the moderational analysis. The findings suggest a moderational effect that might enhance the ICT adoption. These results are of interest in the field of personal development and training purposes in life-span.

## Introduction

Society today has to face constant changes and innovations in technology. Recent studies have evaluated the cognitive impact of devices, which are accessible to the average citizen, such as mobile devices (Dufau et al., 2011; Wilmer et al., 2017). However, the adoption process and its effects are much more complex and sensitive for certain age groups. In recent years there have been numerous investigations carried out linking age to the adoption of ICTs. As a result of these studies, the concept of “digital divide” or digital immigrants among others has emerged. This difference between younger and older adults seems to be especially relevant with respect to mobile devices (Roque and Boot, 2016), although it is also present in the management of computers and the internet (Boot et al., 2013b).

In this way, the notion of a digital divide is not as homogenous as it might seem (Peter and Valkenburg, 2006). Some authors consider that the digital divide is one more expression of the social differences existing in a given territory (Van Dijk and Hacker, 2003).

From this perspective, the weight of sociodemographic characteristics is underlined. Other experts, however, argue that the digital divide is a consequence of relations between citizens and technologies (Anderson et al., 2001; Compaine, 2001).

From this last perspective, it is interesting to review the role that psychological variables – such as cognitive and personality variables – can play as mediators/moderators of this digital divide. In this way, it is not surprising that various ICT adoption models have been developed, such as the Technology Acceptance Model (TAM, Davis, 1989) as well as the Unified Theory of Acceptance and Use of Technology (UTAUT, Venkatesh et al., 2003).

The range of possibilities within this field is vast, where the relationship that exists between the use of ICT with aspects such as perceived usefullness, perceived ease of use, attitude, intention to use, perceived risk and trust is highlighted (Lorenzo Romero et al., 2011). In recent studies this model has been extended to include related variables such as ICT anxiety, and ICT teaching self-efficacy (Mac Callum et al., 2014).

One of the most popular models that includes cognitive variables would be the one described by the CREATE research group (Czaja et al., 2006) on Factors Predicting the Use of Technology on Aging. Their research pointed out that the older adults were less likely than younger adults to use technology in general. Nevertheless, they found some predictors of the use of technology, such as computer anxiety, fluid intelligence, and crystallized intelligence. Therefore, the relationship between age and the adoption of technology was mediated by cognitive abilities, computer self-efficacy, and computer anxiety.

However, there remain underlying questions to specific variables related to attitudes, emotions and values that accompany cognitive processes, such as the cognitive emotions described in the literature (Perkins et al., 1993). Following this same line of relevance of cognitive and personality variables in the use of ICT, but specifically in the elderly population, recent studies support this issue, to the extent that there are authors who propose the new concept of the psycho-digital divide. Indeed, research seems to show that variables of a psychological nature – such as cognitive age, technological anxiety or the level of audacity – can be more explicative of the behavior of the elderly in relation to the use of communication technologies (ICT) than socio-demographic variables are (Peral-Peral et al., 2015).

Other studies also coincide in relating certain personality variables and the use of ICTs. Thus, in the study by Rauschnabel et al. (2015), openness was found be an important personality trait in predicting the use of ICTs, whilst high levels of neuroticism were negatively related to ICT adoption. In the research carried out by Lu et al. (2005), structural equation analysis revealed strong causal relationships between social influences, personal innovativeness and perceptual beliefs—usefulness and ease of use- in the relationship with the intention to adopt ICTs.

A key aspect that is receiving a lot of attention in the psychological field is thinking dispositions. Traditionally, it has been thought that good thinking has been formulated in terms of cognitive ability or skill: “B*eing a good thinker means having certain sorts of critical and creative thinking abilities*” (Tishman and Andrade, 1996). Even if skill thinkers might have these abilities, they also have at their disposal other variables such as attitude, motivation, habits of mind and values. More precisely, all these variables might play a crucial role in good thinking. These components might affect whether people use their thinking abilities when need it. In order to address high-level thinking, both researchers and professionals have paid attention to what are often called “*thinking dispositions*.” Generally speaking, thinking dispositions might be defined as “*tendencies toward patterns of intellectual behavior*” (Perkins et al., 1993). Salomon (1994) described a disposition as a “*cluster of preferences, attitudes, and intentions, plus a set of capabilities that allow the preferences to become realized in a particular way*.” Facione et al. (1994) defined a thinking disposition as a constellation of attitudes, intellectual virtues, and habits of mind.

One of the virtues of thinking that has hardly been investigated in relation to ICT but that we hypothesize that may have a relationship with the use of them is intellectual humility.

Humility can be conceptualized in a number of ways, including as a virtue, a developmental achievement, or an individual trait (Krumrei-Mancuso, 2016). Although humility is often classified as a virtue (e.g., Exline and Geyer, 2004), virtues can have an intellectual rather than moral dimension (Baehr, 2011). From this perspective, Intellectual Humility (IH) can be classified as an epistemic virtue that promotes being a good knower (e.g., Paul and Elder, 2001; Brady and Pritchard, 2003; Stafford, 2010). According to Krumrei-Mancuso, and Rouse (2016), Intellectual Humility can be defined “as a non-threatening awareness of one’s intellectual fallibility” (p.210).

We think that this awareness of intellectual fallibility can play an important role in facing intellectual challenges and overcoming frustration, a key aspect in the adoption of ICT. In this way, IH was associated with underlying variables to openness, curiosity, tolerance of ambiguity, and low dogmatism (Leary, et al., 2017). Moreover, the effect of IH may be also applicable to recognition and memory tasks, having a result in performance and uncertainty (Deffler et al., 2016). These may be an issue of interest in the field of ICT adoption.

An especially interesting aspect of Intellectual Humility in relation to the present study is that IH calls for openness to new ideas (Gruppen, 2014; McElroy et al., 2014) and openness to changing one’s viewpoint when warranted (Hopkin et al., 2014). If the role of this variable in ICT adoption is confirmed, this result could have repercussions in training programs. We bear in mind the importance of this on a daily basis. In this way, the objective of this study is to examine the role of IH in the adoption process of ICT, which is understood as involving two of the most common devices used by the average citizen – mobile phones and computers. In sum, it is expected that the variable under study might moderate the adoption process.

## Materials and Methods

### Participants

A sample of 306 participants (209 women, 68.30%, and 97 men, 31.70%) volunteered to participate in the study. The sampling was incidental. All of them were recruited from the Valencia Community. A number of 20 trained research assistants participated in the data recruitment. The age range had values from 18 to 87 years, M_ean_ = 41.06; *SD* = 18.74. They were also divided into four age groups described as follows: 45 (14.71%) young (18–20 years), 107 (34.97%) adults (21–40 years); 114 (37.25%) middle-aged (41–59 years) and 40 (13.07%) older participants (60–87 years). An incidental sampling method was used. With regards to education, 31.9% were students, 52.4% active workers, 10.4% unemployed, and 11.4% retired. Furthermore, education percentages were composed as follows: 4.45% without studies, 13% elementary studies, 22.1% high school, 49.75% University studies, and 10.7% professional studies. All participants were of legal age (over 18 years). Finally, participants with cognitive impairment or any neurological deficit were not included in the study.

### Instruments

#### Intellectual Humility

The CIHS (Comprehensive Intellectual Humility Scale) is made up of 22 items (Krumrei-Mancuso and Rouse, 2016) with four subscales: (1) independence of intellect and ego (2) openness to revising one’s viewpoint, (3) respect for others’ viewpoints, and (4) lack of intellectual overconfidence. Items were rated on a 5-point Likert scale, ranging from strongly disagree to strongly agree.

The findings in the development of this instrument indicated that the full scale and four factors had satisfactory internal consistency (0.70 or higher), the analyses provided evidence of appropriate levels of construct, convergent, and discriminant validity.

CPQ (Computer Proficiency Questionnaire) (Boot et al., 2013b; Moret-Tatay et al., 2018a). This is a scale to measure computer proficiency and involves factors such as computer basics, printing, communication, Internet, calendaring software, and multimedia use. In terms of internal consistency it reached optimal values (Cronbach’s α = 0.98).

Mobile Device Proficiency Questionnaire (MDPQ) (Roque and Boot, 2016; Moret-Tatay et al., 2018a). This instrument assesses the mobile device proficiency of older adults.

The MDPQ asks participants to rate their ability to perform 46 operations on a smartphone or tablet device. The reliability of this scale was excellent (Cronbach’s α = 0.99).

### Procedure

Participants complete self-administered questionnaires and a written informed consent. In a document it was explained the nature and objectives, ensuring anonymity and confidentiality and emphasizing the honesty of the answers to maximize the validity of the data. The participants took about 30 min to complete the entire set of measures and retained the right to participate or withdraw at any time during the research.

This study was carried out in accordance with the recommendations of the Universidad Católica de Valencia San Vicente Mártir committee’ with written informed consent from all subjects. All subjects gave written informed consent in accordance with the Declaration of Helsinki. This research was approved by the University Ethical Committee with the following number: UCV2017-2018-28.

### Design

The statistical analysis was performed using SPSS 22 (IBM). We conducted a moderational analysis using Process macro for SPSS (Hayes, 2015) to test the hypothesis that intellectual humility moderates the effect of age on the use of mobile devices and computers. In this way, Regression-based moderation procedures were executed employing bootstrapping procedures using 10000 samples (MacKinnon and Fairchild, 2009; Hayes, 2017; Moret-Tatay et al., 2018b).

## Results

As some literature has pointed out that differences between men and women might occur (Liu and Guo, 2017), descriptive analysis were carried out in both sexes as well as the whole dataset. Nevertheless, this was not the case, as depicted in Table 1.

**Table 1:** Descriptive analysis for the variables under study in terms of sex.

	Men Mean	SD	Women Mean	SD	Total Mean	SD
Age	43.887	19.0114	39.82	18.53	41.11	18.75
MDPQ	33.14	9.72	32.80	9.10	32.91	9.29
CPQ	24.70	6.93	24.06	7.29	24.26	7.18
Independence of intellect and ego	21.0928	3.88395	19.97	5.07	20.32	4.75
Openess to revising one’s viewpoint	17.3299	4.22720	15.64	4.79	16.18	4.68
Respect for other’s viewpoint	23.4330	4.44154	23.32	4.81	23.35	4.69
Lack of intellectual overconfidence	23.2680	5.08780	24.15	4.43	23.87	4.66

Secondly, the relationship between variables was examined. The Pearson coefficient was calculated among the variables of interest (see Table 2).

**Table 2:** Pearson coefficient under the variables under study.

	Age	MDPQ	CPQ	Independence	Openess to	Respect for	Lack
				of intellect	revising one’	other’s	intellectual
				and Ego	s viewpoint	viewpoint	overconfident ce
Age	1	-0.748**	-0.693**	0.049	-0.196**	-0.222**	-0.093
MDPQ	-0.748**	1	0.924**	0.041	0.202**	0.199**	0.105
CPQ	-0.693**	0.924**	1	0.099	0.177**	0.165**	0.138*
Independence of intellect and ego	0.049	0.041	0.099	1	0.026	0.184**	0.357**
Openess to revising one’s viewpoint	-0.196**	0.202**	0.177**	0.026	1	0.467**	0.032
Respect for other’s viewpoint	-0.222**	0.199**	0.165**	0.184**	0.467**	1	0.123*
Lack of intellectual overconfidence	-0.093	0.105	0.138*	0.357**	0.032	0.123*	1

*A regression analysis (after a stepwise regression) on the prediction of MDPQ (mobile device proficiency questionnaire) and CPQ (Computer Proficiency Questionnaire) was carried out. The first one (MDPQ) was predicted by Age (β = -0.75; *p* < 0.001) and Independence of intellect and Ego (β = 0.08; *p* < 0.005): *F*(2,304) = 197.15; *p* < 0.001; *R*^*2*^ = 0.56. The second one (CPQ) was also predicted by Age (β = -0.70; *p* < 0.001) and Independence of intellect and Ego (β = 0.13; *p* < 0.05): *F*(2,304) = 149.63; *p* < 0.001; *R*^*2*^ = 0.49. ^∗^Correlation is significant at the 0.05 level, ^∗∗^Correlation is significant at the 0.01 level*.

Finally, a moderational model was carried out. First of all, all factors from intellectual humility were tested as moderators of the relationship between age and use of mobile devices.

**FIGURE 1:**
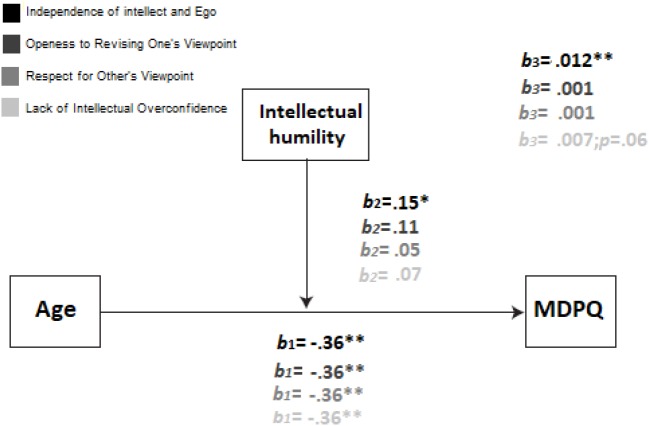
Estimated model, where IH moderate the relationship between age and adoption of mobile devices. X (age) on Y (Mobile adoption) relationships were tested in different values of Z (IH). This model was replicated for the three subscales of Intellectual Humility. B1, B2, and B3 represent the different paths in the model under study. Note that Independence of intellect and ego was the unique model that fulfilled all the assumptions.

The Independence of intellect and Ego reached the statistical significance: *F*(3,301) = 149.45; MSE = 36.61; *p* < 0.001; *R*^2^ = 0.58. The *R*^2^ increased due to the interaction in the model depicted: *F*(1,301) = 9.41; *p* < 0.001; *R*^2^ = 0.01. The model for Openess to Revising One’s Viewpoint also reached the statistical significance: *F*(3,301) = 107.15; MSE = 35.81; *p* < 0.001; *R*^2^ = 0.57. However, not all variables inherent to the model did, as depicted in Figure 1. Furthermore, this case was similar for Respect for Other’s Viewpoint: *F*(3,301) = 103.15; MSE = 38.04; *p* < 0.001; *R*^2^ = 0.56. Once again, the moderational variable did not reach the statistical significance, nor did the interaction. Finally, the model for Lack of Intellectual Overconfidence was statistically significant: *F*(3,301) = 121.88; MSE = 37.82; *p* < 0.001; *R*^2^ = 0.56. The moderational variable did not reach the significance level, as depicted in Figure 1, where the *R*^2^ increased due to the interaction in the model was: *F*(1,301) = 3.45; *p* < 0.06; *R*^2^ = 0.004.

**FIGURE 2:**
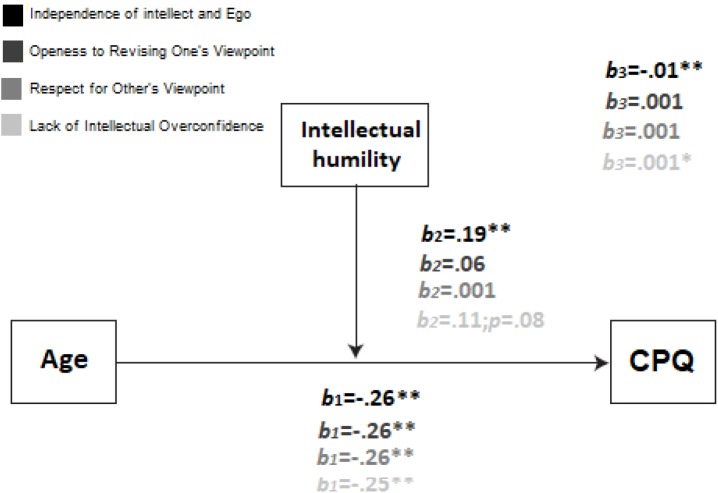
Estimated model, where IH moderate the relationship between age and adoption of computers. X (age) on Y (computer adoption) relationships were tested in different values of Z (IH). This model was replicated for the three subscales of Intellectual Humility. B1, B2, and B3 represent the different paths in the model under study. Note that Independence of intellect and ego was the unique model that fulfilled all the assumptions.

The model was also examined for the Cpq score. In this way, the Independence of intellect and Ego reached the statistical significance: F(3,301) = 113.36; Mse = 25.14; p < 0.001; R^2^ = 0.51. The R^2^ increased due to the interaction in the model depicted: F(1,301) = 12.95; p < 0.001; R^2^ = 0.02. The model for Openess to Revising One’s Viewpoint also reached the statistical significance: F(3,301) = 81.17; Mse = 26.87; p < 0.001; R^2^ = 0.48. However, even if the model was statistically significant, the moderation was not (as previously occurred in the prediction of Mdpq), as depicted in Figure 2. This case was similar for Respect for Other’s Viewpoint: F(3,301) = 78.91; Mse = 26.91; p < 0.001; R^2^ = 0.48. Once again, the moderational variable did not reach the statistical significance, nor did the interaction. Finally, the model for Lack of Intellectual Overconfidence was statistically significant: F(3,301) = 93.38; Mse = 26.21; p < 0.001; R^2^ = 0.49. The moderational variable approached the significance level, as depicted in Figure 1. The R^2^ increased due to the interaction in the model depicted: F(1,301) = 6.04; p < 0.05; R^2^ = 0.01.

## Conclusion and Discussion

The main conclusions can be described as follows: (i) IH might have a moderational role in the adoption process of mobiles and computers, (ii) The main factor in moderation was Independence of intellect and Ego and not other IH constructs (iii) even if MDPQ and CPQ were not correlated with Independence of intellect and Ego, a predicted model reached the statistical significance, stating the predictor independence (iv) Age was also not correlated with Independence of intellect and Ego. Nevertheless, a moderation effect was found as before.

The current study includes an innovative variable, the IH, in the field of adoption of ICT. This variable seems to be a promising one in the study of positive social interactions. Moreover, this variable is also associated with other constructs, such as greater open-mindedness, tolerance and reparation and damaged social bonds (Krumrei-Mancuso, 2016). Both ICT adoption and IH have several points in common, in particular, these variables have a crucial role in personal relationships. The literature has pointed out that older people might accept the challenge on adopting such uncertainty as the ICT offers, to keep in contact with their relatives, among others (Charness and Boot, 2009; Boot et al., 2013a). Moreover, other fields are of interest, such as telehealth among others (Czaja et al., 2013). These researches have highlighted the role of cognitive and attitudinal barriers. In the current study, the focus of attention includes processes related to both of them, such as thinking disposition, and more precisely, IH. Furthermore, this variable may be invariant to aging, as most of the personality variables are.

Moderation usually indicates that the effect of X (age) on Y (ICT adoption) is different for different values of Z (IH). In this way, IH moderates or affects the relationship between age and ICT adoption, a robust relationship in the literature. On a methodological level, predictors can be related to an association in several ways, allowing researchers to interpret the underlying relationship, that may vary across fields. Moreover, both predictors are considered independent, but interact in the prediction of the Y variable. The lack of previous correlations might indicate this as well, discarding also multicollinearity problems.

On the other hand, specifically in relation to the independence of the intellect and the ego, reference is made to the fact that a person can have an open mind and not feel offended by sharing beliefs which are different to their own. Together, the items refer to the fact that when someone contradicts personal important beliefs, they do not take it as a personal attack. People who value positive social relationships may have this factor, which is a prominent aspect in the adoption of ICTs. In turn, intellectual humility also means accepting more challenges and being more tolerant of frustration, which could derive from the learning of ICT.

Some limitations have been found. First of all, women are overrepresented, as commonly found in this type of studies. In this way, it is crucial to highlight that the recruitment procedure was incidental, so, different bias might occur.

Considering that intellectual humility is a disposition of thought, some authors (Ennis, 1987; Perkins, 1995) have supported the idea that thinking dispositions can be taught. This highlights the application of the current results in terms of training. Furthermore, Perkins in “*Outsmarting IQ: The emerging science of learnable intelligence*,” examines some piece of research regarding several programs for teaching thinking. Furthermore, he highlights some evidence for the learnability underlying the dispositional tendencies, reviewing skills programs as Odyssey, Instrumental Enrichment, CoRT, and Philosophy for Children. Finally, Perkins determines that intelligence can be learned in certain ways: e.g., how to be more reflective, to seek more alternatives, to provide more explanations and reasons, and to be more imaginative. Literature has also shed light on the benefits across life span, and these gains are reflected in modest improvements in IQ scores.

Therefore, we believe that promoting these types of intellectual virtues could suppose a protective effect that cushions the effects of age on adaptability to new times. Future lines of research must examine the role of IH as a protective factors related to social changes.

## Author Contributions

GB-V conceived and designed the study, collected and analyzed the data, and wrote the manuscript. II-M participated in the revision of the literature, study design, data collection and analysis, and drafting of the manuscript. MB-A and NS-C designed the study, collected the data, and revised the literature.

## Conflict of Interest Statement

The authors declare that the research was conducted in the absence of any commercial or financial relationships that could be construed as a potential conflict of interest.
